# Transition and Life‐Long Care for Adults With Cerebral Palsy: A Patient Group ‘Too Hard to Impact!’ Are We Still Sending Young People ‘Off a Cliff’?

**DOI:** 10.1111/jep.70011

**Published:** 2025-02-28

**Authors:** Susie Turner, Charlotte Nash, Jane Goodwin, Johanna Smith, Charlie Fairhurst, Jill Cadwgan

**Affiliations:** ^1^ Evelina London Children's Hospital, Guy's and St Thomas’ NHS Foundation Trust London UK; ^2^ Mathematical Institute, University of Oxford Oxford UK; ^3^ Population Health Sciences Institute, Newcastle University Newcastle upon Tyne UK

**Keywords:** cerebral palsy, quality of health care, transitional care, young adults

## Abstract

**Objectives:**

At Evelina London Children's Hospital, tertiary care is provided for children with Cerebral Palsy (CP) across the South East of England. An increasing number of adults with CP remain under the care of the children's hospital due to a lack of appropriate adult neurodisability services. This quality improvement project as part of a gap analysis regarding transition pathways for young adults with CP, aimed to explore the lived experience of young adults within our service, with respect to transition and care in adulthood.

**Methods:**

This was a multi‐methods design. Firstly, a bespoke questionnaire to young adults with CP and their families evaluated their experience of transition, access to services, challenges with care, and their needs from healthcare professionals (*n* = 46). Then, a focus group with parents of adults with CP (*n* = 4) and interviews with adults with CP (*n* = 5) informed design of an ideal transition pathway and adult service. The qualitative data were thematically analysed.

**Results:**

Framework analysis of the questionnaire data was mapped against the International Classification of Functioning (ICF) with the following themes identified: Body structure and function: pain and anxiety; Activity: equality; accessibility and relationships; Participation: need for friendship and social opportunities, employment and education; Environmental factors: health services and providers; home environment and wider community; Personal factors: independence. Analysis of the focus group and interviews identified five main themes: Gradual and co‐ordinated transition process; Co‐ordination of care in adult services; Knowledge, skills and experience of professionals; Communication; and Worrying about the future.

**Conclusions:**

Participants identified significant challenges with transition pathways and adult care. Annual reviews from healthcare professionals with expertise in CP should be offered to young adults to ensure early identification of health needs. Further research is needed to support business planning in development of appropriate adult services for adults with CP and ensure successful transition pathways.

## 1 Introduction

Cerebral Palsy (CP) is a lifelong condition with a UK prevalence of 1–2 per 1000 [1]. Previously considered a childhood condition, with advancements in neonatal and paediatric care, many more children with CP now survive into adult life and can have a similar life expectancy to the general population [2]. Individuals with CP have multiple and complex co‐morbidities, especially pain, with subsequent impact onto their quality of life [3, 4].

The National Institute for Care and Health Excellence (NICE) guideline and quality standard on ‘Transition from children's to adults’ services for young people using health or social care services’ provides evidence‐based guidance on how transition should happen and highlights specific areas for improvement [5, 6]. In addition, the NICE guidelines ‘Cerebral palsy in under 25 s: assessment and management’ and ‘Cerebral palsy in adults’ provide further evidenced based recommendations in supporting this patient group [7, 8].

Despite this, transition from child to adult services has been poorly managed in CP [9], lacking both planning and documentation [10]. Continuing access to adult services, especially for those with complex co‐morbidities, remains challenging [11]. The period of transition is often associated with deterioration in the health of adolescents with chronic conditions [12]. For example, many young people with CP do not access physical therapy during and postsecondary school, partly due to difficulties accessing disability support services [13]. Failure of the transition pathway means many individuals with CP and their families continue to rely on paediatric services or end up discharged to primary care services, with little or no specialty service input [14, 15]. This can affect participation, functional mobility, and quality of life [13].

According to NICE guidance on CP [7] it is standard practice for all children with cerebral palsy in the UK to have support from a local multidisciplinary team with referrals to tertiary neurodisability and other specialist services according to co‐morbidities. In addition, the Cerebral Palsy Integrated Pathway (CPIP) is a patient management programme for children aged 2 to 16 years with a diagnosis of CP. This was formally introduced in the SE region in 2018. Each individual on the database receives a standardised assessment and review by a CPIP trained physiotherapist of their musculoskeletal system and participation. Timings of assessments and hip x‐rays for surveillance are based on Gross Motor Function Classification System (GMFCS) levels [16]. The assessment results guide changes in management and indicate when referrals to orthopaedics and tone management services are needed. Both longitudinal and cross‐sectional studies have shown the importance of hip surveillance for children with cerebral palsy at detecting hip displacement early, enabling earlier surgeries and a reduction in the prevalence of CP hip dislocation [17, 18].

**Aim:**

This quality improvement project was part of a gap analysis of transition pathways and adult service provision for individuals with CP in the South East (SE) region. This paper summarises the findings from the young adults with CP and their families' perspective.

**Objectives:**

1. To survey (via questionnaire) young adults with CP and their families regarding their:
  a. current access to services in adulthood compared to services provided in paediatric services
  b. satisfaction with their experience of transition from paediatric to adult services
2. To collect further qualitative information from young adults with CP and their families regarding their:
  a. perceptions of current health and social care provision and impact on their life across International Classification of Functioning (ICF) domains [19].
  b. Vviews about changes required to provide their ideal adult service.

## 2 Methods

### 2.1 Participants

A population of young adults in the SE region of the UK, known to our regional service, were sampled for this study:

1. Young adults with CP and their families (questionnaire).
2. Parents of young adults with severe CP and intellectual disability (focus group).
3. Young adults with CP (interviews).

### 2.2 Measures

A questionnaire was devised following a literature review, and consultation with young adults, parents and child health professionals. The questions included the demographic characteristics of respondents and their experience accessing support, their symptoms (e.g., location of pain), and satisfaction with transition. Respondents could skip any questions they did not want to answer. The survey was hosted online through the patient experience tool: ‘Civica Experience’ via Guy's and St Thomas' NHS Foundation Trust. Participants provided their email addresses and the link to complete the online survey was sent via email. The questionnaire enabled participants to choose which questions were relevant for them to answer and this was overt from the outset.

After the questionnaire responses had been thematically analysed, the results were summarised to share with focus group and interview participants, as an introduction. The focus group and interview participants were then asked to use a solution focussed approach; and the topic guides included questions on what changes were needed to provide their ideal transition and adult service and essential skills required from health care professionals. Both the interviews and focus group were purposively sampled and participation was voluntary.

### 2.3 Ethics

This gap analysis was a registered audit at Evelina London Children's Hospital and as a service evaluation, this did not require ethical approval (based on HRA Decision‐Making Tool: (http://www.hra-decisiontools.org.uk/research/). All participants were informed about the study, introduced to the research team and gave informed consent before taking part. Additionally, participants have provided consent for publication of these findings.

### 2.4 Procedure

This is part of a wider gap analysis which included a survey of health‐care professionals—their knowledge of NICE guidance and Quality Standards for children and adults with CP and transition and their existing transition pathways [20]. Recruitment was in the SE region of England and took place between November 2020 and April 2021. Reminder emails were sent 2 weeks and 4 weeks later. For the interviews and focus group, participants received information about the project during clinical appointments and were followed up by their lead clinician to see if they had any questions if they wanted to take part. Following consent, focus group participants received a meeting invite and interviews were arranged at a time convenient for the participant. As this was during the Covid‐19 pandemic, the focus group and the interviews were conducted online and lasted for an hour. Audio and visual recordings were made. The focus group, interviews and transcripts were carried out by a research psychologist (JG) who has worked within our service, but was not part of the participants direct clinical care team. Participants have been informed of the main findings of the study.

### 2.5 Analysis

Quantitative data analysis was descriptive, largely reporting percentages of respondents in each category for each question. Open text responses from the questionnaires were analysed thematically with Framework Analysis [21] based on the ICF [19]. The interviews and focus group were recorded and transcribed. Three authors independently analysed the data using thematic analysis [22]. The themes identified were discussed and agreed by all authors.

### 2.6 Patient and Public Involvement

Before data collection at each stage: the content of the questionnaire, the introduction and topic guides for focus groups, and interviews; pilot feedback was obtained from young adults, their families and health care professionals regarding the content and ease of use. A parent advisor (JS) was involved in the process from onset, supporting questionnaire design, data analysis; and the focus group and interview topic guides. She attended the focus group as parent‐carer support and is a co‐author of this manuscript.

## 3 Results

### 3.1 Survey of Young People With CP and Their Families

#### 3.1.1 Participants

A total of 46 questionnaires were returned from 56 sent out to young adults and their families known to our service, resulting in a response rate of 82%, demonstrating a high level of engagement. Table 1 outlines the respondent characteristics. Most participants were female and family members/carers. 73.9% of the young people were dependent on a wheelchair to mobilise.

**Table 1:** Demographics of participants who completed the questionnaire for young adults with CP and their families.

Person answering the questions	*N* (%)
Carer/family member	12 (26.1)
Young person with CP	7 (15.2)
Young person with CP and carer together	5 (10.9)
Other	1 (2.2)
Unanswered	21 (45.6)
Sex	
Female	17 (37.0)
Male	7 (15.2)
Unanswered	22 (47.8)
Sexual orientation	
Heterosexual	23 (50.0)
Prefer not to say	1 (2.2)
Unanswered	22 (47.8)
Age group	
16–18	6 (13.0)
19–25	9 (19.6)
26–29	3 (6.5)
30 and over	7 (15.2)
Unanswered	21 (45.6)
Ethnicity	
White British	22 (47.83)
Other^a^	2 (4.35)
Prefer not to say	1 (2.17)
Unanswered	21 (45.7)
Mobility level	
Unable to walk—use of wheelchair required	34 (73.9)
Able to walk short distances independently and use a wheelchair for longer distances	3 (6.5)
Able to walk independently	4 (8.7)
Unanswered	5 (10.9)

^a^ Ethnicity provided but not stated to avoid identifying the participants.

#### 3.1.2 Survey Results

53.3% of participants had not transitioned due to lack of accessible adult services, despite reporting they should now be under adult services and they remained under the care of paediatric service(s). The majority of young people were accessing a GP in the borough they lived in and had less than 5 contacts with them in the past year. Only 6.5% of participants were satisfied or very satisfied with their experience of transition, compared to 32.6% who were dissatisfied or very dissatisfied. 58.7% of participants stated they were accessing a specialist who reviewed their overall health and had access to tone management services. Further, free‐text responses demonstrated that the majority of the specialist services accessed were paediatric with many comments from participants about the challenges of accessing many different adult specialist services highlighted.

Only 2.2% of participants reported their overall care in adult service was better than in paediatrics compared to 23.9% stating it was worse. See Table 2.

**Table 2:** Young people's access to services.

Attending education in the borough they live	*N* (%)
Yes	9 (19.6)
No	12 (26.0)
Not applicable	20 (43.5)
Unanswered	5 (10.9)
Accessing a case worker	
Yes	21 (45.7)
No	19 (41.3)
Unanswered	6 (13.0)
Accessing a GP in the borough they live	
Yes	40 (87.0)
No	1 (2.1)
Unanswered	5 (10.9)
Contact with the GP in the past year	
None	10 (21.8)
Once	8 (17.4)
2–5 times	15 (32.6)
6–9 times	6 (13.0)
10+ times	1 (2.2)
Unanswered	6 (13.0)
Accessing a specialist who reviews overall healthcare	
Yes	27 (58.7)
No	6 (13.0)
Sometimes	4 (8.7)
Don't know	2 (4.4)
Unanswered	7 (15.2)
CPIP assessment whilst in children's services	
Yes	10 (21.8)
No	18 (39.1)
Don't know	10 (21.7)
Unanswered	8 (17.4)
Specialist services being accessed^a^	
Tone management	27 (58.7)
Neurology	18 (39.1)
Orthopaedics	17 (37.0)
Dietitian	17 (37.0)
Epilepsy	12 (26.1)
Spinal orthopaedics	10 (21.8)
Gastroenterology	8 (17.4)
Respiratory	7 (15.2)
ENT	7 (15.2)
Pain team	6 (13.0)
Mental health services	6 (13.0)
Urology	5 (10.9)
Women's health	2 (4.4)
Rehabilitation team	1 (2.2)
Palliative care	1 (2.2)
Unanswered	7 (15.2)
Are you able to access the services required when needed?	
Yes, always	5 (10.9)
Yes, sometimes	5 (10.9)
No	1 (2.2)
Unanswered	35 (76.1)
Do you/your young adult have access to orthotics if required?	
Yes, always	2 (4.3)
Yes, sometimes	7 (15.2)
No, but I would like this	9 (19.6)
No, not required	17 (37.0)
Unanswered	11 (23.9)
Thinking about the period of transition from children's to adult's care—overall how satisfied or dissatisfied were you with the process?	
Very satisfied	2 (4.3)
Satisfied	1 (2.2)
Neither satisfied or dissatisfied	5 (10.9)
Dissatisfied	7 (15.2)
Very dissatisfied	8 (17.4)
Don't know	2 (4.3)
Unanswered	21 (45.7)
Thinking about the overall care you/your young adult currently receives under the care of adult services in comparison to what you/they received under the care of children's services. How do adult services compare to children's services?	
Better	1 (2.2)
Same	5 (10.9)
Different	7 (15.2)
Worse	11 (23.9)
Unanswered	22 (47.8)

^a^ Participants could select more than one answer; therefore, the total number of responses are greater than the number of participants. Percentages are calculated out of the number of participants.

34.8% of Participants reported that they did not have access to a social worker (and would like to); 23.9% had not had wheelchair review within the last year (even though they would like to). See Table 3.

**Table 3:** Young people's needs related to support services.

	Seeing a physiotherapist either through an education placement or in your local community?	Seeing a Social Services Occupational Therapist?	Seeing a Healthcare Occupational Therapist?	Seeing a Speech and Language Therapist for communication needs?	Seeing a Speech and Language Therapist for eating and drinking needs?	Has wheelchair been reviewed within the last year?	Using augmentative and alternative communication?	Receiving support from carers (paid or voluntary)?	Receiving support from a social worker?	Access to respite?
Yes	19 (41.3)	10 (21.7)	8 (17.4)	11 (23.9)	8 (17.4)	21 (45.6)	9 (19.6)	—	15 (32.6)	—
Right amount	—	—	—	—	—	—	—	23 (50.0)	—	7 (15.2)
Need more	—	—	—	—	—	—	—	10 (21.7)	—	8 (17.4)
No, but I would like to	5 (10.9)	2 (4.4)	10 (21.7)	7 (15.2)	3 (6.5)	11 (23.9)	1 (2.2)	1 (2.2)	16 (34.8)	8 (17.4)
Don't know	2 (4.4)	—	—	—	—	—	—	—	—	—
No, not required	4 (8.7)	4 (8.7)	4 (8.7)	11 (23.9)	19 (41.3)	5 (10.9)	2 (4.3)	4 (8.7)	6 (13.0)	15 (32.6)
I'm not seeing them at the moment but I can access this if I need to	6 (13.0)	19 (41.3)	13 (28.3)	7 (15.2)	6 (13.0)	—	—	—	—	—
Missing	10 (21.7)	11 (23.9)	11 (23.9)	10 (21.8)	10 (21.8)	9 (19.6)	34 (73.9)	8 (17.4)	9 (19.6)	8 (17.4)

52.2% of young people reported experiencing difficulty accessing support from their GP. See Table 4.

**Table 4:** Young people's challenges accessing services.

Difficulty accessing needs via GP	*N* (%)
Yes, always	8 (17.4)
Yes, sometimes	16 (34.8)
No	17 (36.9)
Missing	5 (10.9)
Remaining under child services even though they should be in adult services	
Yes	24 (52.2)
No	16 (34.8)
Missing	6 (13.0)

Open‐ended responses from the questionnaire were analysed using the ICF framework [21]. A summary is provided below.

Diagram A shows a summary of comments regarding experiences of transition mapped against the ICF framework [19].

#### 3.1.3 Body Structure and Function

Two main themes were emergent: (1) pain—respondents reported pain which ranged from mild to severe, as impacting onto daily life: *‘My pain levels stop me enjoying and engaging with social occasions’* [Young person, aged 24]; *‘more pain management’* [Young person, aged 20]; and (2) Anxiety: Participants reported anxiety, both for themselves as well as for family members: *‘I have not got a carer to take me out in the evening as there are difficulties finding carers who are able to deal with my complex needs. I get incredible bored at home and get frustrated and upset as I rely on my mum to do everything for me. My mum is exhausted and provides full care as well as dinners, housework and double the amount of laundry than normal households, she is also trying to keep me out and about and entertained. It is putting strain on her’*. [Young person, aged 19]. *‘…. I feel uncomfortable and at times embarrassed or judged by others. I stay mostly within the confines of my home where I can manage my mobility’*. [Young person, aged 22]. Anxiety about hospital admissions under adult services was reported: ‘*Expectations that I will be able to stay in a hospital on my own without my support’* [Young person, age 18].

**DIAGRAM A:**
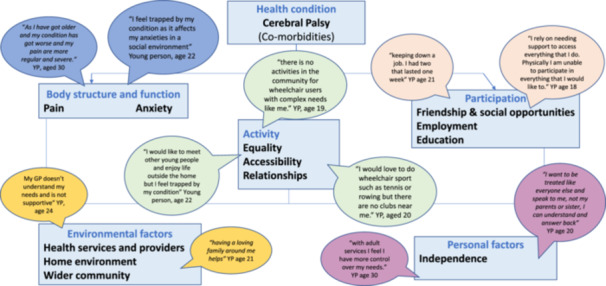
Experiences of transition mapped against the ICF [19, 20].

#### 3.1.4 Activity

Three main themes were noted: (1) Equality *‘I would like a local disco or club to be available for people like me once a week.’* [Young person, aged 18]; (2) Accessibility *‘I would like to be able to attend holiday club, go out in the evening more and do activities that are inclusive for wheelchair users’* [Young person aged 19]; ‘*Swimming somewhere that is accessible & convenient for me & my family’* [Young person aged 20]; (3) Relationships *‘Level of physical disability acts as a barrier to accessing others unless it is in a fully accessible environment. Few opportunities to make friends’*. [Parent of young person, aged 24]. Lack of opportunity to develop independent relationships *‘Currently rely on mum to sort social life … and then she has to come with us too*’ [Young person, aged 21].

#### 3.1.5 Participation

Participation had three main themes: (1) need for friendship and social opportunities *‘I would like to be able to meet people at clubs or social events so I could make friends’* [Young person, aged 21]; *‘As a young adult there are no social activities other than the Day centre that I can attend because of my age and disabilities’* [Young person, aged 20]; *‘It would be good to have a life outside of the home and direct family’* [Young person aged 22]; *‘As going out becomes more and more taxing it's become more and more discouraging and has led to me barely leaving the house. I still speak to friends online but my social circle has shrunk a lot’* [Young person aged 22]; *‘No clubs or social activities set up for accessing as a young adult’* [Young person aged 18]; *‘Not a lot to do ‐ very few clubs or places my daughter can go’* [Parent of young person aged 17]; and (2) employment *‘I'm working on employment at home, whereby I don't let an employer down’* [Young person aged 21]; *‘I use self‐teaching tutorial in finance to fill my days’;* [Young person age 20]; and (3) education ‐ respondents identified impact of illness on education. *‘My son has not been fit to attend school and in the last 4 months he has been in hospital 6 times. We would like a solution to his stomach issues to get him back to school’* [Parent of young person aged 19].

#### 3.1.6 Environmental Factors

Three main themes were emergent: (1) health services and providers: participants reported that a clear pathway with trusted professionals was necessary, along with individualised care and communication between services *‘Communication and contact would be handy…. we really feel like we've been pushed off a cliff with transition.’* [Parent of a young person aged 18]; *‘Lack of communication with the new adult services. From having all the support in children's services to virtually nothing!’* [Parent of young person aged 20]; *‘There is no care available for me. I don't see anybody from adult services’* [Young person aged 25]. Difficulty accessing appropriate support from GPs was highlighted ‘*GP is pleasant but their role cannot match the oversight of a Paediatrician and they don't have the expertise or knowledge to be able to address complex needs.’* [Parent of young person aged 19]; *‘GP always seems worried to see my daughter due to having CP’* [Parent of YP age 18].

*‘My daughter needs Gynaecology support but the GP didn't refer her. I had to sort the support privately’* [Parent of young person aged 19]; *‘Investigating complaints of health issues by GP has been problematic’* [Parent of young person aged 19]; Accessibility at GP surgeries was identified as an issue due to manual handling difficulties in offering clinical examinations as needed: *‘GP surgeries do not have hoists so it is not possible to do physical examinations’* [Parent of young person aged 19]; as well as difficulties accessing the GP surgery *‘Parking space may not be available’* [Parent of young person aged 24]; (2) home environment: accessibility and family relationships were most important; *‘I stay mostly within the confines of my home where I can manage my mobility, I have lots of support from family’* [Young person aged 21]; and (3) wider community: participants reported that accessibility and education were a priority. *‘Lack of facilities in the community. Think we are in the ‘too hard to make an impact’ group’*. [Parent of young person aged 18]. Barriers reported included: *‘Lack of wheelchair access to locations/disabled toilets’ and ‘most activities for disabled people are sport which is no good for me as I have no movement’* [Young person aged 19].

#### 3.1.7 Personal Factors

The main theme identified was independence: participants needed to develop their autonomy and self‐advocacy in age appropriate services *‘with adult services I feel I have more control over my needs.’* [Young person, aged 30]; ‘*Driving my car on my own without my parents being with me.’* [Young person aged 25]; Participants highlighted the importance of this during care giving ‘*gives me independence and they respect my space’* [Young person, aged 24].

### 3.2 Focus Group With Parents and Interview With Young People With CP

#### 3.2.1 Participants

We invited young adults known to our service to represent a range of motor severity (GMFCS), co‐morbidities, gender, age, and communication methods.

Four parents of young adults with CP GMFCS level V and intellectual disability (ages 18–20) participated. Five young adults with CP GMFCS levels II, III & IV (ages 20–31) took part in the interviews. One has mild intellectual disability, but was able to self‐advocate. None of the others had intellectual disability.

#### 3.2.2 Focus Group and Interview Findings

Five main themes were identified during analysis and are outlined below.

### 3.3 Gradual and Coordinated Transition Process

The need for a graduated and coordinated transition process to provide continuity of care was identified. Participants reported on the importance of continuing support from a known healthcare professional whilst establishing a relationship with adult healthcare professionals. Importantly, a key healthcare professional to contact for advice and support would reduce a feeling of abandonment.

A transition period, when you kind of–your child services and you adult services communicate or work together for a small period of time, just to get to know you and to really know what you need in place……. and sometimes it takes a while for people to get to know the context. But I think if people already had somebody there that could sort of do an extended handover, it might be more of a smooth transition.

(Young person, aged 27)

Consistency in seeing the same healthcare professional was identified. They recognised that time is needed to develop trust and relationships with new healthcare professionals, providing opportunity for healthcare professionals to engage with and understand the young person's abilities:

I think the most important thing is to have some continuity. You know, if you go to see a specialist and then you go the next time and you see someone completely different, and they're quickly trying to scrabble through the notes and say ‘oh this was done last time’ and unless you happen to remind them of something and they say ‘oh it wasn't mentioned in the notes’ and they go back a bit ‘oh yes it was’ and they're not familiar with your child—your young adult at all.

(Parent of young person, aged 20)

I'm lucky I can talk, but others can't… I think they need to be able to be a person's friend as well as a person's professional. Because it then puts whoever they're talking to at ease and makes them feel like they can talk about anything or relax or just be themselves. Trust is a big thing.

(Young person, aged 31)

### 3.4 Co‐Ordination of Care in Adult Services

Accessing support from adult services was challenging:

there is a complete lack of information in the paediatric service of who to transition our children to in adult services……it's been really difficult to get our lead paediatrician to find out who to transition us to. And getting equipment and things like that… so we're still getting them from children's services, because nobody, including the GP, the district nurses, the learning disability nurses or anybody else knows who to transition us to.

(Parent of young person, aged 18)

Individual paediatric services transition young people at different ages which adds to confusion about the process:

…. the whole transition side is so confusing to me. Everybody does it at different stages at different times, you know, so some people have held onto us, some people have let us go, and it's very–I'd like a clear—at this point they transition instead of higgledy‐piggledy everywhere.

(Parent of young person, aged 19)

The ability to develop a community network with others living with CP was described as a missed opportunity.

Sometimes… don't really understand how I shower or how I cut my vegetables, or how I butter my bread. But someone with a disability could relate and be like ‘try this! Or try that’.

(Young person, aged 27)

Participants felt as though they were fighting for services:

When my son needs to see a specialist it's a mission to get referred anywhere.

(Parent of young person, aged 20)

The importance of having an annual review was highlighted:

we need that one person—even if you see them once a year just to go, ‘this is going well, this is going well, I haven't seen this person, I need a referral to this person’, just so we've got that contact. I think that one main person still needs to happen into adulthood.

(Parent of young person, aged 19)

### 3.5 Knowledge, Skills, and Experience of Professionals Involved in Their Care

All participants identified a lack of understanding, knowledge, and expertise about CP from adult healthcare professionals they encountered, including GPs. This led to mistrust in healthcare professionals and a reluctance to move on from children's services.

there's no, you know, understanding in GP either about CP. So if you go to the GP and you're like ‘my whole body aches’—because of the CP, they're not going to understand the CP to the level that you understand it. You have to explain it to them…

(Young person, aged 20)

Understand the different types of CP… Everyone's movement is different around the varieties of CP. It's not all the same, they need to have a better understanding of that. If you're going to a professional for the first time and they have no clue what CP is, how are you supposed to get the benefit out of it?

(Young person, aged 20)

Our GP has been copied into every single letter we've had in the last 8 years, but it doesn't qualify him to be able to help X refer to adult services. I mean he's a general practitioner, he knows a little bit about everything but he doesn't know all about X, I don't think he's even met her. So how is he supposed to know what I'm talking about when it's taken me 18 years to learn what I'm talking about, so I don't think the GP is the best person to be the lead consultant for my daughter…

(Parent of young person, aged 18)

### 3.6 Communication

Participants highlighted the need for healthcare professionals to improve their communication skills with the young people with CP and/or their families.

Having services that will listen to you. Because I know that sometimes when I have been to the doctors and hospitals and stuff, sometimes they don't let me explain how I'm doing and what limitations I have in terms of that specific thing. They will just be like ‘oh let's do this and let's do that’ and I'm not able to express fully the condition that I'm in that day and the service I need from them.

(Young person, aged 27)

Healthcare professionals need to acknowledge that the young person is the expert in their own life:

I don't like people telling me how to live my life……because you're not living it, you don't really understand what I'm going through, so be a human. Don't be a doctor, because… I understand where you're coming from, but it's a lot harder for me to do those when you're not doing them yourself and you don't know the day to day ins and outs.

(Young person, aged 27)

Communicating in nonmedical terminology was a barrier: it is the healthcare professional's role to meet the young person's needs, which allows for mutual understanding.

I want someone who is human, who will come down to my level, where I can understand them and they understand me. And then we progress.

(Young person, aged 27)

### 3.7 Worrying About the Future

All parents expressed concern about the future and the impact on them as well as their children due to the reduction in support following transition:

I just think that without these services, we all tend to have the same opinion that our kids are just going to be home now, you're going to have a load of burned‐out parents very soon. As much as we're doing it, we can't keep doing it forever.

(Parent of young person, aged 19)

I used to be able to get up all night and go to work the next day. Now if I'm up in the night, it wipes me out the next day. So, we can't keep on doing it.

(Parent of young person, aged 20)

I'm expecting to do less for my daughter because she's getting older, so as a parent I'd like that as well. But it's now the opposite.

(Parent of young person, aged 19)

Parents highlighted they worry about their ability to recognise when to ask for help if they don't have access to healthcare professionals with expertise:

I feel like the responsibility is on us to know when we need a service and as much as we know our kids, we're not trained professionals.

(Parent of young person, aged 19)

…. I have no idea what to look out for, or what's serious, or what's not serious. And again, to put that back on myself to think ‘ok I really need to make sure I get a referral to the kidney people because I don't feel that's right’—well, I don't know if that's right or not. So, I think having someone just to help with that, would be really, really, really helpful, and just easing some of that anxiety which is possibly totally unfounded, but I don't know. I feel like as a parent, you don't want to miss something which further down the line you could have prevented just because you didn't know anything about it.

(Parent of young person, aged 19)

Young people highlighted their awareness of the impact on their parents:

Important to me as well is that my family and my mum gets the support they need and there's someone my family can go to if they've got any problems, because often my mum worries about stuff and how I'm going to be looked after but there doesn't seem to be anywhere at all.

(Young person, age 21)

## 4 Discussion

### 4.1 Statement of Principle Findings

We aimed to understand from the perspective of young people with CP and their families their experience of transition and access to services in adulthood. Through analysing the questionnaire results it became clear the majority of the young adults had not been able to transition successfully to adult services due to lack of availability. In a solution focused approach, we explored young adults and parents/carers views on what changes are needed to provide their ideal adult service.

Participants in the survey, focus group, and interviews highlighted transition remains a challenging and unsatisfactory time for young people with CP. Experiences of pain, anxiety, and inequity of accessing health and social care services were reported. The lack of coordination of the process, lack of a key healthcare professional to contact and difficulties accessing appropriate support from GPs were highlighted. In particular, the lack of access to an adult healthcare professional who understands CP was strongly identified as a barrier in supporting and meeting their needs in adult services. Once young people transition into adult services there is no clear subspecialty clinician to take over responsibility for their holistic care and frequently this responsibility falls to GPs. NICE recommends ‘adults with cerebral palsy who have complex needs have an annual review with a healthcare professional with expertise in neurodisabilities.’ [23]. The existing NICE guidance clearly sets out how transition and access to support in adult services should look [5, 7, 8]. Despite these guidelines, there remains a lack of adult service provision to support young people with CP once they leave paediatric services and this may be placing additional burden on GPs. Therefore, reflection on the structure of adult services, and which healthcare professional groups are best placed to support adults with CP, is needed to enable better understanding of this gap in provision of services. Without appropriate adult healthcare services to transition young people with CP onto then challenges with transition pathways and accessing support in adult services will continue.

The paediatric CPIP surveillance programme commenced in the SE region in 2018. Therefore, the low number of participants in this study who were part of CPIP would be expected given the majority, due to their age, would have been out with the age range. In addition, many areas across the region started registering the younger age groups onto the pathway first.

In Sweden, the CP pathway was extended lifelong to include adults with CP and facilitates lifelong follow up [24]. Given the difficulties with transition and access to adult services identified in the current study, extending this pathway to include adults in the UK could provide the opportunity for a meaningful review from healthcare professionals with expertise in CP.

### 4.2 Strengths and Weaknesses of the Study

The excellent response rate obtained in this project, is related to PPI feedback regarding the length of questionnaire enhanced engagement from the participants, as they were re‐assured that they did not need to complete the whole questionnaire. The qualitative design and use of focus group and interviews enabled in‐depth insights from the participants on the need for development of adult services to improve transition pathways in CP.

The participants however, sampled from our regional paediatric service that continues to support an increasing number of young adults with CP due to a lack of appropriate adult neurodisability services, results in a selection bias, and is likely to be an underestimate of the needs of individuals with CP in the wider community, or other regions in the UK.

In the questionnaire, there were a large proportion of missing data about demographics. As participants were known to the service they may have been concerned about identification leading to missing data, especially on demographics. Additionally, none of the questions were mandatory and along with the length of the survey this may also have contributed to missing data. Participants may have been more interested in sharing their ideas, and thus focused their energy on these questions rather than more routine demographic information.

Data were collected during the first year of the covid‐19 pandemic, which meant that potential participants were difficult to access and challenges with services may have been exacerbated for some people during this time.

### 4.3 Strengths and Weaknesses in Relation to Other Studies

Previous studies have highlighted the complexities of transition for young people with cerebral palsy in comparison to other long‐term health conditions, and need for commissioning in children's and adult services [25, 26]. but there has been limited research to identify the reality of current service provision for adults with CP in the UK, particularly since the publication of the NICE guidance [8, 23].

### 4.4 Meaning of the Study: Possible Explanations and Implications for Clinicians and Policy Makers

This project, as part of a wider gap analysis of provision for adults for cerebral palsy, will influence imminent change needed for regional services, has contributed to recommendations for national health policy [27], and provides clear steps for further research. It shows the importance of individuals with CP and their families having trust in the expertise of health care professionals they access across the course of their lives, and strongly supports the need for training and education for professionals, including GPs to enable this.

### 4.5 Unanswered Questions and Future Research

Future research should collaboratively work with young adults with CP to codesign appropriate health and social care services for adults with CP, as without this there can be no transition pathway. Further, as GPs were identified as the main contact for adults with CP, understanding GPs' perspectives on their experiences of supporting adults with CP would provide insight into challenges and potential solutions.

## Author Contributions

Susie Turner is responsible for the overall content as guarantor. She contributed to all aspects from study conception to final approval of the manuscript. Jill Cadwgan and Charlie Fairhurst contributed to the design, analysis, and interpretation of the work. Charlotte Nash, Jane Goodwin, and Johanna Smith contributed to the analysis and reporting.

## Conflicts of Interest

The authors declare no conflicts of interest.

## Transparency Statement

Susie Turner affirms that the manuscript is an honest, accurate, and transparent account of the project being reported; that no important aspects of the project have been omitted; and that any discrepancies from the project as originally planned (and, if relevant, registered) have been explained.

## Data Availability

The data that support the findings of this study are kept on NHS confidential server. The authors can be contacted if further information is required. Relevant data is available upon reasonable request.
